# Alzheimer’s disease progression by geographical region in a clinical trial setting

**DOI:** 10.1186/s13195-015-0127-0

**Published:** 2015-06-25

**Authors:** David B Henley, Sherie A Dowsett, Yun-Fei Chen, Hong Liu-Seifert, Joshua D Grill, Rachelle S Doody, Paul Aisen, Rema Raman, David S Miller, Ann M Hake, Jeffrey Cummings

**Affiliations:** Eli Lilly and Company, Indianapolis, IN 46285 USA; Indiana University School of Medicine, Indianapolis, IN 46202 USA; University of California, Irvine, Institute for Memory Impairments and Neurological Disorders, 3206 Biological Sciences III, Irvine, CA 92697-4545 USA; Baylor College of Medicine, Department of Neurology, Houston, TX 77030 USA; University of California San Diego School of Medicine, 9500 Gilman Drive, La Jolla, CA 92093-0717 USA; Dementia and Geriatric Psychiatry, Bracket, 575 E. Swedesford Rd, Ste 200, Wayne, PA 19087 USA; Cleveland Clinic Lou Ruvo Center for Brain Health, 888 W Bonneville, Las Vegas, NV 89106 USA

## Abstract

**Introduction:**

To facilitate enrollment and meet local registration requirements, sponsors have increasingly implemented multi-national Alzheimer’s disease (AD) studies. Geographic regions vary on many dimensions that may affect disease progression or its measurement. To aid researchers designing and implementing Phase 3 AD trials, we assessed disease progression across geographic regions using placebo data from four large, multi-national clinical trials of investigational compounds developed to target AD pathophysiology.

**Methods:**

Four similarly-designed 76 to 80 week, randomized, double-blind placebo-controlled trials with nearly identical entry criteria enrolled patients aged ≥55 years with mild or moderate NINCDS/ADRDA probable AD. Descriptive analyses were performed for observed mean score and observed mean change in score from baseline at each scheduled visit. Data included in the analyses were pooled from the intent-to-treat placebo-assigned overall (mild and moderate) AD dementia populations from all four studies. Disease progression was assessed as change from baseline for each of 5 scales - the AD Assessment Scale-cognitive subscale (ADAS-cog11), the AD Cooperative Study- Activities of Daily Living Scale (ADCS-ADL), Mini-Mental State Examination (MMSE), the Clinical Dementia Rating scored by the sum of boxes method (CDR-SB), and the Neuropsychiatric Inventory (NPI).

**Results:**

Regions were heterogeneous at baseline. At baseline, disease severity as measured by ADAS-cog11, ADCS-ADL, and CDR-SB was numerically worse for Eastern Europe/Russia compared with other regions. Of all regional populations, Eastern Europe/Russia showed the greatest cognitive and functional decline from baseline; Japan, Asia and/or S. America/Mexico showed the least cognitive and functional decline.

**Conclusions:**

These data suggest that in multi-national clinical trials, AD progression or its measurement may differ across geographic regions; this may be in part due to heterogeneity across populations at baseline. The observed differences in AD progression between outcome measures across geographic regions may generalize to 'real-world' clinic populations, where heterogeneity is the norm.

**Trial registrations:**

ClinicalTrials.gov NCT00594568 – IDENTITY. Registered 11 January 2008.

ClinicalTrials.gov NCT00762411 – IDENTITY2. Registered 26 September 2008

ClinicalTrials.gov NCT00905372 – EXPEDITION. Registered 18 May 2009

ClinicalTrials.gov NCT00904683 – EXPEDITION2. Registered 18 May 2009

## Introduction

Alzheimer’s disease (AD) is a progressive neurodegenerative disorder generally first manifesting as cognitive impairment, progressing to impairment of daily function and, ultimately, loss of independence, debility, and death from complicating medical comorbidities. In the past decade, researchers have studied treatments that target the underlying pathophysiology of AD, as yet without approval of any disease-modifying drug entity. Development of such disease-modifying therapies is made challenging by the length and size of studies required to demonstrate positive effects on co-primary outcome measures of cognition and function. Most trials have enrolled several hundred to more than a thousand patients, with studies lasting 12 to 18 months [1]. In order to enroll patients in a reasonable time period and meet regulatory requirements for local registration, sponsors have increasingly implemented more multinational AD studies [2] that cover multiple geographic regions and encompass many cultures, languages, and healthcare delivery systems. Multinational AD studies may be helpful in furthering our understanding of the effects of a therapy across various standards of care, family structures, and societal views on outcomes [3].

Despite this trend for large, multinational AD trials, relatively little is known about the implications of conducting them [3]. There are multiple reasons to expect heterogeneity in these trials. Making a clinical diagnosis of AD is challenging and, historically, has occurred by elimination of other potential etiologies. Even in the clinical trial setting, 18 to 22% of patients clinically diagnosed with AD were found to be lacking evidence of pathophysiology of AD using amyloid positron emission tomography tracers [4,5]. Moreover, AD is a complex disease with multiple risk factors including advancing age, lower education level, and carrying the ε4 allele of the apolipoprotein E (*APOE*) gene as well as other specific genetic loci [6-10]. Differences in prevalence of risk factors, variability in clinical diagnosis and differences related to culture, access to healthcare, and clinical trial conduct across geographic regions [2] may result in heterogeneity in patient populations recruited for global AD trials. Additional factors that could lead to heterogeneity in multinational AD trials were discussed at a meeting of representatives from the Alzheimer’s Association, sponsors, regulatory bodies, and vendors, and were published by Doody and colleagues [3]. This heterogeneity has the potential to result in differences in rates of disease progression across regions. While differences in dementia prevalence across geographic regions have been documented [11], to date there is a paucity of published data on disease progression across geographic regions.

To aid researchers in designing, implementing, and analyzing data from multinational phase 3 AD trials, we assessed disease progression across geographic regions using placebo data from four large, multinational clinical trials of compounds developed to target the underlying pathophysiology of AD [12,13]. An additional perspective on AD across geographic regions is provided by Grill and colleagues [14], who assessed recruitment, retention, and safety reporting across regions using data from these four AD trials.

## Methods

Placebo data from four randomized double-blind placebo-controlled AD trials (IDENTITY, IDENTITY2, EXPEDITION, EXPEDITION2) were used in this exploratory analysis. The study designs have been published previously [12,13,15,16]. Study protocols were reviewed and approved by the relevant ethical review boards (see Acknowledgements). Briefly, IDENTITY and IDENTITY2 were 76-week trials designed to study the effect of semagacestat, a γ-secretase inhibitor no longer in development, on the progression of AD; EXPEDITION and EXPEDITION2 were 80-week trials designed to study the effect of solanezumab, a humanized anti-Aβ peptide antibody currently in development, on the progression of AD. For each trial, the research protocol was approved by the ethical review board at each study site participating in that trial. Written informed consent for study participation was provided by the study subject or a legally authorized representative, in accordance with the Declaration of Helsinki. The current study analyzing data collected across these clinical trials was reviewed by the University of California, Los Angeles (UCLA) Medical Institutional Review Board and deemed as not meeting the definition of human subjects research.

The entry criteria were nearly identical for the four studies and included patients 55 years and older with moderate or mild AD dementia, documented on the basis of a score of 16 to 19 and of 20 to 26, respectively, on the Mini-Mental State Examination (MMSE), and meeting criteria of the National Institute of Neurological and Communicative Diseases and Stroke–Alzheimer’s Disease and Related Disorders Association for probable AD. Patients with other etiologies for dementia were to be excluded.

Subjects were required to be medically stable with a reliable study partner who spent >10 hours per week with the patient. Subjects were permitted to receive cholinesterase inhibitors and/or memantine during the studies but had to be stable in dose prior to entry and remain stable during the studies.

Efficacy measures in the four studies included the 11-item Alzheimer’s Disease Assessment Scale – cognitive subscale (ADAS-cog11; range 0 to 70, higher scores worse) [17], the Alzheimer’s Disease Cooperative Study – Activities of Daily Living Scale (ADCS-ADL; range 0 to 78, lower scores worse) [18], the Clinical Dementia Rating scored by the sum of boxes method (CDR-SB; range 0 to 18, higher scores worse) [19,20], the MMSE (range 0 to 30, lower scores worse) [21], and the Neuropsychiatric Inventory (NPI; range 0 to 144, higher scores worse) [22,23]. Scales were translated into the native language(s) of the region and the ADAS-cog11 and ADCS-ADL were administered by raters trained and qualified in their administration and scoring. Training without qualification was provided for all other scales administered in the trials. The protocols specified that the same rater was to rate the ADAS-cog11 throughout the study and this rater should not rate the ADCS-ADL. If a rater left a site, both training and qualification of the new rater was required. Raters falling below minimal pretrial experience levels in administering the ADAS-cog were required to complete additional (enrichment) training and pass a prequalification examination before undergoing the formal qualification training and examination at the startup meeting. In addition, if raters were incorrectly scoring the ADAS-cog11 or MMSE as determined during instudy rating reviews (performed at baseline and 52 weeks for the IDENTITY program, and at baseline and 12 weeks for the EXPEDITION program), they were contacted and reminded of the correct scoring algorithm and asked to correct their error(s).

IDENTITY and IDENTITY2 were implemented at 300 sites in 31 countries, with enrollment from April 2008 to May 2010. EXPEDITION and EXPEDITION2 were implemented at 211 sites in 16 countries, with enrollment from May 2009 to June 2012. As the result of identifying an unfavorable benefit/risk ratio with semagacestat in an interim safety analysis, the IDENTITY studies were amended to discontinue the study drug and follow study subjects for an additional 7 months. Only placebo data from the initial, randomized study period of up to 76 weeks were considered in the present analyses. At the time at which the IDENTITY protocols were amended, both studies were fully enrolled with 37.7% and 6.1% of the IDENTITY and IDENTITY2 study subjects, respectively, having been followed for the full 76-week initial study period [15]. The EXPEDITION and EXPEDITION2 studies were completed in April 2012 and June 2012 with 73% and 78% of the study populations, respectively, observed for the full 80 weeks.

### Statistical analysis

The small sample sizes in most countries (Table 1) necessitated regional instead of by-country analyses. Geographic regions were defined based on a modified version of criteria used by Glickman and colleagues [24]. Countries were combined into regions based on ethnicity and healthcare delivery systems, to increase sample size. Regions were as follows: North America (United States, Canada); South America/Mexico (Argentina, Brazil, Chile, Mexico); Western Europe/Israel (Belgium, Denmark, Finland, France, Germany, Israel, Italy, Spain, Sweden, United Kingdom); Eastern Europe/Russia (Bulgaria, Hungary, Poland, Romania, Russia, Serbia, Turkey, Ukraine); Australia/South Africa; Asia (China, India, Korea, Taiwan); and Japan.

**Table 1:** **Number of sites, enrolled subjects and subjects completing the study by country and region**

**Region/country**	**Number of sites**	**Number enrolled**	**Completers,** ***n*** **(%)** ^**a**^	
			**EXPEDITION**	**IDENTITY** ^**b**^
**North America**	**179**	**832**	**325 (72)**	**140 (37)**
United States	154	714	286 (71)	127 (41)
Canada	25	118	39 (80)	13 (19)
**Western Europe/Israel**	**112**	**412**	**163 (77)**	**71 (36)**
Belgium	4	8	–	5 (63)
Denmark	2	5	–	3 (60)
Finland	3	6	–	6 (100)
France	16	69	23 (92)	9 (20)
Germany	23	104	42 (76)	14 (29)
Israel	7	22	–	8 (36)
Italy	18	74	42 (74)	1 (6)
Spain	16	48	15 (63)	12 (50)
Sweden	12	37	23 (85)	8 (80)
United Kingdom	11	39	18 (72)	5 (36)
**South America /Mexico**	**50**	**196**	**82 (75)**	**8 (9)**
Argentina	25	88	43 (67)	0
Brazil	14	67	39 (85)	0
Chile	7	17	–	8 (47)
Mexico	4	24	–	0
**Eastern Europe/Russia**	**49**	**195**	**38 (61)**	**5 (4)**
Bulgaria	5	18	–	1 (6)
Hungary	4	16	–	1 (6)
Poland	13	48	24 (67)	3 (25)
Romania	4	17	–	0
Russia	11	45	14 (54)	0
Serbia	3	8	–	0
Turkey	4	23	–	0
Ukraine	5	20	–	0
**Japan**	**46**	**191**	**71 (88)**	**23 (21)**
**Asia**	**40**	**169**	**59 (84)**	**0**
China	6	22	–	0
India	7	10	–	0
Korea	16	85	33 (79)	0
Taiwan	11	52	26 (93)	0
**Australia/South Africa**	**25**	**84**	**32 (84)**	**18 (39)**
Australia	18	58	32 (84)	10 (50)
South Africa	7	26	–	8 (31)

^a^For the EXPEDITION program, a completer was defined as a subject who had completed the 80-week double-blind study period. For the IDENTITY program, a completer was defined as a subject who had completed the 76-week initial treatment period; the denominator in this case was the number of subjects who had opportunity to complete 76 weeks of treatment before the study drug was stopped at request of the sponsor, and the study was amended.

^b^Several countries were included only in the IDENTITY program (Belgium, Denmark, Finland, Israel, Chile, Mexico, Bulgaria, Hungary, Romania, Serbia, Turkey, Ukraine, China, India and South Africa).

Analyses were performed using SAS version 9.2 (SAS Institute Inc, Cary, NC). Descriptive analyses were performed for the observed mean score and the observed mean change in score from baseline at each scheduled visit. Spearman’s rank correlations were used to assess the relationship among AD scales at baseline and among baseline-to-endpoint (18 months) changes in scores by region. Data included in the analyses were pooled from the intent-to-treat placebo-assigned overall (mild and moderate) AD populations from all four studies. Disease progression was assessed as the change from baseline for each of the four scales (ADAS-cog11, ADCS-ADL, MMSE, CDR-SB). Measurements considered in the analyses were those performed at baseline and 76/80 weeks (depending on study), as well as at 12, 28, 40, 52, and 64 weeks for ADAS-cog11 and ACDS-ADL, 52 weeks for MMSE, and 28 and 52 weeks for CDR-SB.

For the EXPEDITION program, a completer was defined as a subject who had completed the 80-week double-blind study period. For the IDENTITY program, a completer was defined as a subject who had completed the 76-week initial treatment period; the denominator in this case was the number of subjects who had an opportunity to complete 76 weeks of treatment before the study drug was stopped at request of the sponsor and the study was amended.

## Results

### Enrollment and study completion by region and country

Subject enrollment and completion is shown by country and region in Table 1. Overall, data from 2,079 subjects assigned placebo (EXPEDITION, *n* = 506; EXPEDITION2, *n* = 519; IDENTITY, *n* = 501; IDENTITY2, *n* = 553) were included in the analyses. Since the study drug was stopped before intended study termination in the IDENTITY program and the studies were amended, many study subjects did not have the opportunity to participate until the endpoint visit at 76 weeks. As a result, the IDENTITY program had a numerically smaller proportion of completers than the EXPEDITION program.

### Baseline characteristics by region

**Figure 1:**
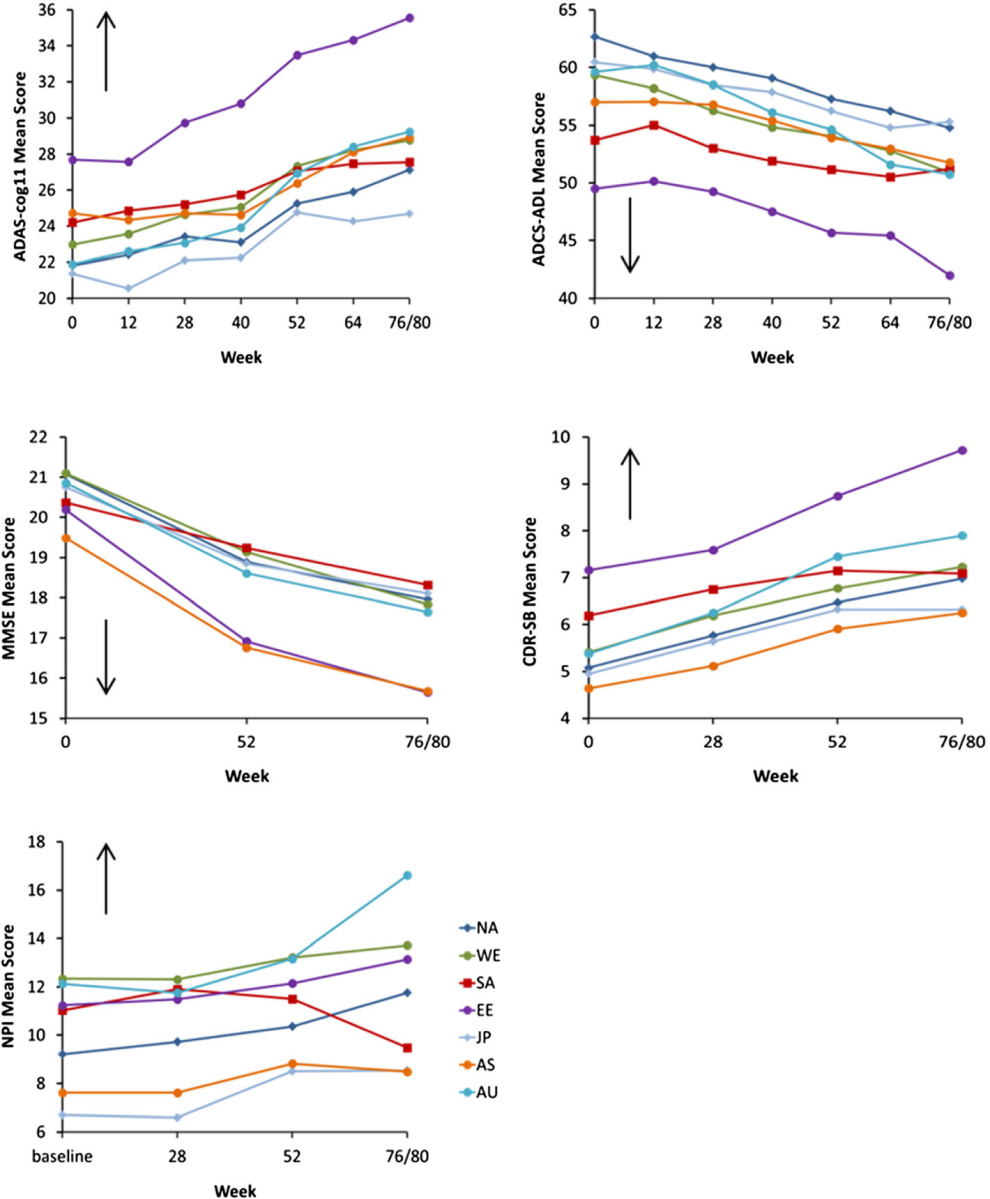
**Observed mean score at each time point by region for outcome measure scores.** ADAS-cog11, 11-item Alzheimer’s disease Assessment Scale – cognitive subscale; ADCS-ADL, Alzheimer’s disease Cooperative Study – Activities of Daily Living; CDR-SB, Clinical Dementia Rating Scale sum of boxes; MMSE, Mini-Mental State Examination; NPI, Neuropsychiatric Inventory.

There were numerical differences in baseline characteristics among regions for these placebo-assigned subjects (Table 2). The Asia population had the lowest proportion of subjects with mild disease, defined as MMSE 20 to 26 (42%); generally, subjects were oldest in North America and South America/Mexico, and youngest in Western Europe/Israel and Eastern Europe/Russia; subjects had received the most years of education in North America and least education in South America/Mexico and Asia; there were fewer males than females enrolled overall, but Western Europe/Israel enrolled the highest and South America/Mexico and Eastern Europe/Russia the lowest proportions of male subjects; while 74 to 94% of subjects received concomitant AD treatment, it was most common in Western Europe/Israel and least common in Eastern Europe/Russia and Australia/South Africa; and *APOE* ε4 carriers were most common in Western Europe/Israel and North America and least common in Asia. Baseline disease severity, as measured by ADAS-cog11, ADCS-ADL, and CDR-SB, was worse for Eastern Europe/Russia compared with populations in other regions (Table 3, Figure 1), but this was not the case for the MMSE and NPI.

**Table 2:** **Baseline characteristics by geographic region**

	**North America**	**Western Europe/Israel**	**South America**	**Eastern Europe/Russia**	**Japan**	**Asia**	**Australia/South Africa**	**Overall**
	**(** ***n*** **= 832)**	**(** ***n*** **= 412)**	**(** ***n*** **= 196)**	**(** ***n*** **= 195)**	**(** ***n*** **= 191)**	**(** ***n*** **= 169)**	**(** ***n*** **= 84)**	**(** ***N*** **= 2,079)**
Study population with mild AD	547 (66%)	265 (64%)	114 (58%)	106 (54%)	132 (69%)	71 (42%)	57 (68%)	1,292 (62%)
Mean (SD) age (years)	75.0 (8.1)	71.6 (7.7)	74.6 (8.0)	70.9 (7.7)	73.1 (7.7)	72.2 (7.7)	73.0 (7.1)	73.4 (8.0)
Mean (SD) education (years)	14.0 (3.1)	11. 2 (4.2)	9.1 (4.5)	11.8 (3.7)	11. 8 (2.8)	9.6 (4.7)	12.2 (3.4)	12.1 (4.1)
Male	381 (45.8%)	205 (49.8%)	66 (33.7%)	70 (35.9%)	73 (38.2%)	68 (40.2%)	38 (45.2%)	901 (43.3%)
AChEI and/or memantine	736 (88.5%)	387 (93.9%)	167 (85.2%)	145 (74.4%)	173 (90.6%)	139 (82.2%)	66 (78.6%)	1,813 (87.2%)
*APOE* ε4 carriers	481 (63.0%)	218 (65.7%)	96 (51.1%)	90 (51.4%)	98 (53.0%)	39 (42.4%)	51 (61.4%)	1,073 (59.0%)
Mean (SD) baseline scores								
ADAS-cog11	21.81 (9.01)	22.97 (9.18)	24.20 (8.75)	27.69 (11.13)	21.37 (6.80)	24.72 (7.72)	21.89 (9.51)	23.02 (9.16)
ADCS-ADL	62.68 (11.66)	59.34 (13.62)	53.72 (14.17)	49.51 (16.91)	60.45 (11.16)	57.02 (4.80)	59.62 (13.04)	59.16 (13.76)
MMSE	21.08 (3.67)	21.10 (3.56)	20.37 (3.07)	20.19 (3.15)	20.75 (3.10)	19.49 (3.57)	20.86 (3.49)	20.77 (3.51)
CDR-SB	5.08 (2.48)	5.41 (2.70)	6.19 (2.74)	7.16 (3.34)	4.95 (2.66)	4.64 (2.53)	5.38 (2.32)	5.41 (2.74)
NPI	9.21 (10.94)	11.03 (11.73)	12.34 (12.82)	11.24 (11.86)	6.70 (8.69)	7.62 (8.68)	12.13 (10.80)	9.81 (11.13)

Data presented as number (%) or mean (standard deviation). AChEI, acetylcholinesterase inhibitor; AD, Alzheimer’s disease; ADAS-cog11, 11-item Alzheimer’s disease Assessment Scale – cognitive subscale; ADCS-ADL, Alzheimer’s disease Cooperative Study – Activities of Daily Living; APOE, apolipoprotein E; CDR-SB, Clinical Dementia Rating Scale sum of boxes; MMSE, Mini-Mental State Examination; NPI, Neuropsychiatric Inventory; SD, standard deviation.

**Table 3:** **Observed change from baseline to 76/80 weeks by region for outcome measure scores**

	**North America**	**Western Europe/Israel**	**South America**	**Eastern Europe/Russia**	**Japan**	**Asia**	**Australia/South Africa**	**Overall**
	**(** ***n*** **= 832)**	**(** ***n*** **= 412)**	**(** ***n*** **= 196)**	**(** ***n*** **= 195)**	**(** ***n*** **= 191)**	**(** ***n*** **= 169)**	**(** ***n*** **= 84)**	**(** ***N*** **= 2,079)**
ADAS-cog11								
6 months	1.62 (5.66)	1.99 (6.12)	0.99 (6.51)	3.06 (7.23)	0.68 (5.04)	0.21 (6.06)	1.64 (5.97)	1.55 (6.00)
12 months	3.88 (7.40)	4.92 (7.76)	3.23 (7.28)	6.53 (9.11)	3.18 (6.57)	1.49 (7.04)	5.44 (8.39)	4.07 (7.62)
18 months	6.04 (9.44)	7.46 (9.68)	4.76 (8.41)	10.95 (10.77)	4.41 (7.99)	3.52 (7.98)	7.3 (11.54)	6.23 (9.48)
ADCS-ADL								
6 months	−3.2 (7.45)	−3.41 (8.90)	−1.17 (9.09)	−2.07 (8.63)	−2.03 (6.78)	−2.05 (7.86)	−2.29 (9.82)	−2.71 (8.12)
12 months	−6.26 (10.01)	−6.08 (11.08)	−3.7 (9.79)	−6.39 (12.80)	−4.13 (8.76)	−4.71 (10.02)	−6.18 (13.12)	−5.66 (10.51)
18 months	−9.16 (12.13)	−10.84 (13.44)	−5.57 (12.78)	−11.51 (14.16)	−5.94 (9.39)	−7.85 (9.75)	−9.00 (14.89)	−8.95 (12.48)
MMSE								
6 months	–	–	–	–	–	–	–	–
12 months	−2.29 (3.56)	−2.14 (3.84)	−1.39 (3.49)	−3.48 (4.73)	−1.79 (3.34)	−2.22 (2.92)	−2.45 (3.79)	−2.21 (3.68)
18 months	−3.39 (4.59)	−3.66 (4.70)	−2.52 (4.18)	−5.28 (5.95)	−2.78 (4.13)	−2.93 (4.06)	−3.45 (4.73)	−3.38 (4.60)
CDR-SB								
6 months	5.77 (3.06)	6.19 (3.24)	6.75 (3.18)	7.59 (3.82)	5.64 (3.31)	5.12 (2.74)	6.25 (3.11)	6.05 (3.24)
12 months	6.47 (3.46)	6.77 (3.67)	7.15 (3.47)	8.74 (4.09)	6.32 (3.71)	5.91 (3.24)	7.45 (3.86)	6.75 (3.64)
18 months	6.98 (3.91)	7.23 (3.98)	7.09 (3.59)	9.72 (4.66)	6.32 (3.92)	6.25 (3.25)	7.90 (4.32)	7.10 (3.96)
NPI								
6 months	0.73 (9.52)	0.87 (10.43)	0.00 (11.04)	0.09 (10.79)	−0.05 (6.79)	0.71 (9.47)	0.68 (10.73)	0.55 (9.77)
12 months	1.57 (11.15**)**	0.63 (11.25)	0.81 (13.48)	0.82 (10.88)	2.04 (9.09)	2.02 (12.76)	2.44 (11.46)	1.37 (11.30)
18 months	2.86 (13.36)	2.97 (13.98)	−1.80 (14.08)	2.30 (12.57)	2.41 (8.95)	1.83 (10.00)	5.29 (14.11)	2.47 (13.11)

Data presented as mean (standard deviation). ADAS-cog11, 11-item Alzheimer’s disease Assessment Scale – cognitive subscale; ADCS-ADL, Alzheimer’s disease Cooperative Study – Activities of Daily Living; CDR-SB, Clinical Dementia Rating Scale sum of boxes; MMSE, Mini-Mental State Examination; NPI, Neuropsychiatric Inventory.

### Experience with ADAS-cog11 rating by region

Across study programs, enriched training was required most frequently in Asia and Japan. In IDENTITY, North America, Western Europe/Israel, Eastern Europe/Russia, and Australia/South Africa had less need for remedial training (Table 4); in EXPEDITION, less need for remedial training was evident in Western Europe/Israel, followed by North America and Eastern Europe/Russia.

**Table 4:** **Requirement for enrichment training for those rating ADAS-cog11**

**Trial**	**North America**	**Western Europe/Israel**	**South America**	**Eastern Europe/Russia**	**Japan**	**Asia**	**Australia/South Africa**	**Overall**
	**(** ***n*** **= 832)**	**(** ***n*** **= 412)**	**(** ***n*** **= 196)**	**(** ***n*** **= 195)**	**(** ***n*** **= 191)**	**(** ***n*** **= 169)**	**(** ***n*** **= 84)**	**(** ***N*** **= 2,079)**
IDENTITY	26/167 (16)	25/139 (18)	14/39 (36)	9/54 (17)	20/48 (42)	14/40 (35)	5/28 (18)	113/515 (22)
EXPEDITION	23/145 (16)	8/90 (9)	7/41 (17)	4/30 (13)	16/51 (31)	9/23 (39)	9/23 (39)	76/403 (19)

Data presented as raters in the study who had required enrichment training/total number of raters in the study (%). ADAS-cog11, 11-item Alzheimer’s disease Assessment Scale – cognitive subscale.

### Disease progression by region

Of all regional populations, Eastern Europe/Russia showed the greatest cognitive and functional decline from baseline; Japan, Asia, and/or South America/Mexico showed the least cognitive and functional decline (Table 3, Figure 1). For ADAS-cog11 specifically, Eastern Europe/Russia showed the most cognitive decline over the course of the study (mean change from baseline to 18 months was 11.0) while Asia and Japan showed the least decline (mean change from baseline to 18 months was 3.5 and 4.4, respectively); North America, Australia/South Africa, and Western Europe/Israel showed a similar decline (mean change from baseline to 18 months was 6.0 to 7.5). In the case of the NPI, the 18-month decline was greatest for Australia/South Africa, while there was some improvement in score at 18 months for South America/Mexico.

### Correlations between outcome measures by region

**Figure 2:**
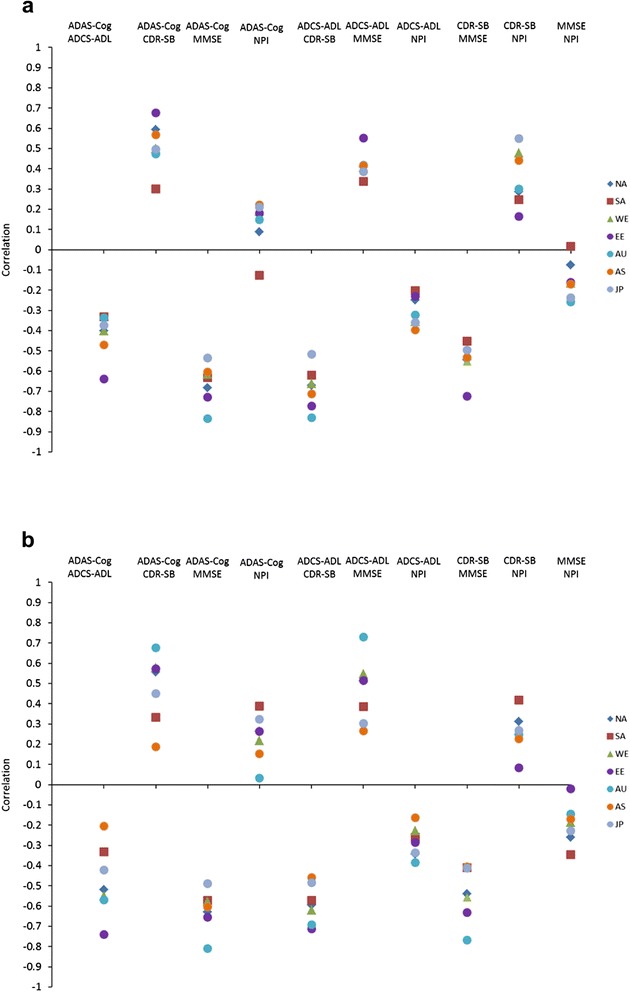
**Correlation between scales by region. (a)** Baseline measures. **(b)** Mean change from baseline to 18 months for study completers. ADAS-cog11, 11-item Alzheimer’s disease Assessment Scale – cognitive subscale; ADCS-ADL, Alzheimer’s disease Cooperative Study – Activities of Daily Living; AS, Asia; AU, Australia/South Africa; CDR-SB, Clinical Dementia Rating Scale sum of boxes; EE, Eastern Europe/Russia; JP, Japan. MMSE, Mini-Mental State Examination; NA, North America; NPI, Neuropsychiatric Inventory; SA, South America/Mexico; WE, Western Europe/Israel.

Correlations among outcome measures at baseline and changes in outcome measures from baseline to endpoint for each region are shown in Figure 2. The range of correlations across regions was generally greater for change from baseline than at baseline. Scales that include cognitive assessment (ADAS-cog11 and MMSE) were consistently well correlated across regions (−0.5 to −0.8); functional (ADCS-ADL) and global (CDR-SB) assessment scale scores were also well correlated across regions. For change from baseline to endpoint, these correlations were generally lowest for Asia and/or Japan and highest for Eastern Europe and/or Australia/South Africa. Measures of cognition (ADAS-cog11 or MMSE) were less correlated with functional (ADCS-ADL) or global scales (CDR-SB). Correlations between NPI scores and other scale scores were generally less than 0.5.

## Discussion

The objective of this analysis was to better understand disease progression among geographic regions in the setting of multinational AD clinical trials, based on analysis of placebo data from the IDENTITY and EXPEDITION study programs. Although some regions had relatively small sample sizes (Australia/South Africa, *n* = 84), differences in AD progression or its measurement over the trial periods were evident across regions. Eastern Europe/Russia showed the greatest cognitive and functional decline from baseline amongst the regions on the ADAS-cog11 and ADCS ADL scales (see Table 3), while Asia, Japan, and South America/Mexico showed the least. The two regions with the largest study populations, North America and Western Europe/Israel, showed a similar decline. For the 18-month change from baseline on the ADAS-cog11, the 11.0 point increase for Eastern Europe/Russia was appreciably different from the regions with the least change from baseline (Asia 3.5 points, Japan 4.4 points). Change from baseline for North America and Western Europe/Israel, as well as Australia/South Africa, ranged from 6.0 to 7.5 points.

The differences among geographic regions observed here may have been the result of heterogeneity in the study populations across regions. Younger age, female gender, greater baseline disease severity, absence of treatment with acetylcholinesterase inhibitors and/or memantine, and an *APOE* ε4 genotype have been associated with more rapid clinical disease progression [6,25,26]. The study population of Eastern Europe/Russia was younger, had a higher proportion of females, and had a lower proportion treated with AD medications at baseline; baseline outcome measure scores were also generally more severe for Eastern Europe/Russia, compared with the other populations. The proportion of the Eastern European/Russian population who were *APOE* ε4 carriers (51%) as well as the proportion with mild AD at baseline (54%), however, lay in the middle of the range across regions. More detailed findings on differences/similarities in baseline characteristics across these geographic regions are presented by Grill and colleagues, who concluded that populations recruited in to clinical trials are likely to differ across regions due to multiple factors – differences in lifestyle factors, overall health, access to medical care, standard of AD diagnosis and treatment, reimbursement for AD services and treatment, family attitudes toward AD recognition, reporting of symptoms and research participation, diagnosis and treatment, and ethnogenetic differences including those resulting in different prevalence of *APOE* ε4 carrier status [14]. Another factor which could contribute to variability in measurement of disease progression in these multinational clinical trials is language differences; although a centralized translation service was used to minimize the effect of translation on outcomes, this still does not guarantee equivalence among cultural groups or regions. Local differences may require slight adjustment of particular items. For example, in the IDENTITY and EXPEDITION study programs, orientation to county on the MMSE had to be adjusted to accept a response of ‘region’ or ‘burro’ where the concept of counties was not applicable. This may have contributed to some variability in outcomes across regions because patients may recall region more readily than county.

Differences in levels of rater experience could also have contributed to the observed differences in measurement of AD progression across regions. Yet in these study programs, extensive rater training – including enriched training where necessary and qualification at investigator meetings – was implemented before raters were permitted to administer the ADAS-cog11. Miller and colleagues demonstrated previously that raters who require and receive enriched training for ADAS-cog11 perform similarly to their more experienced colleagues [27]. Therefore, it is unlikely that rater experience alone could account for the differences in measurement of AD progression seen in our analyses.

Based on the findings from these analyses, it is prudent to be mindful of potential regional differences when designing trials, performing analyses, and interpreting findings, so that information collected is of maximum benefit to all populations who will ultimately have access to the drug entity, once approved. Differences in AD progression or its measurement across geographic regions in the clinical trial setting are probably reflective of the real-world situation where heterogeneity of populations and their treatments is expected and common. Importantly, in the IDENTITY and EXPEDITION programs, the regional differences did not preclude detection of active drug effects [12,13]. If a drug effect can be detected within a study population showing some heterogeneity in disease progression, the effects are more probably generalizable to a heterogeneous clinic population.

For these analyses, we also assessed correlations between scales across regions. Scales that measure cognition (ADAS-cog11 and MMSE) were consistently well correlated with each other across regions but there was more variability among regions in correlations between cognitive (ADAS-cog11 or MMSE) and functional (ADCS-ADL) or global scales (CDR-SB), with some regions showing higher correlations than others. Better understanding of why this variability occurred will require further study, but potential differences among cultures in the relevance of functional measures could have contributed [28]. In addition, the cognitive assessments are performance-based tests administered to the patient, whereas the functional scales are proxy report by the caregiver. Since functional scales are more subjective in nature, these may be more susceptible to cultural influences, and this may contribute to regional variability in correlations between scales.

To our knowledge, this is the first published analysis assessing AD progression in a clinical trial setting across geographic regions. Schneider and Sano reviewed data from 11 AD clinical trials of patients with mild-to-moderate dementia, conducted both in the United States and outside the United States, but did not perform regional analyses [1]. Overall, for these 11 studies the 18-month mean change from baseline on ADAS-cog11 ranged from 4.34 to 9.10 (standard deviation 8.2 to 9.4); analysis methodology did differ across the studies. In the present analyses, overall findings were similar, with an 18-month mean change in ADAS-cog11 of 6.23 (standard deviation 9.48).

There are limitations to these analyses. Geographic groupings – while based on those of Glickman and colleagues [24] and expected similarities in environmental factors (for example, healthcare, culture) across countries within regions – may be somewhat arbitrary, and heterogeneity within regions is likely. Analysis by country would have reduced this effect to some degree, but sample sizes were generally small at the country level, limiting the interpretability of findings. In some countries, patients were enrolled only in IDENTITY, a program in which the study drug was stopped due to an unfavorable benefit/risk ratio for active drug (semagacestat). As a result, the proportion of study completers in these IDENTITY-only countries was small. Despite grouping countries into regions, the sample size limitation remained to some extent and we performed descriptive analyses rather than formal comparisons across regions.

## Conclusion

These data suggest that AD progression or its measurement may differ across geographic regions in multinational clinical trials; this may be in part due to heterogeneity across populations at baseline. The observed differences in AD progression and correlations between outcome measures across the geographic regions may be reflective of the real-world situation, where heterogeneity of populations and their treatments is expected and common. Trial sponsors will need to continue to implement multinational studies due to required study sizes, enrollment rates, and regulatory requirements; these data will be helpful in study planning.

## Abbreviations

AD: Alzheimer’s disease
ADAS-cog11: 11-item Alzheimer’s Disease Assessment Scale –cognitive subscale
ADCS-ADL: Alzheimer’s Disease Cooperative Study – Activities of Daily Living
APOE: apolipoprotein E
CDR-SB: Clinical Dementia Rating Scale sum of boxes
MMSE: Mini-Mental State Examination
NPI: Neuropsychiatric Inventory
